# Supplementary material for: Effect of motivational interviewing intervention on HgbA1C and depression in people with type 2 diabetes mellitus (systematic review and meta-analysis)
Source: PLoS One. 2020 Oct 23;15(10):e0240839. doi: 10.1371/journal.pone.0240839 (PMC7584232; doi:10.1371/journal.pone.0240839)
Supplement: S2 File — (ZIP) [file pone.0240839.s002.zip › Supporting Information files/extracted data.docx]

**Table: Extracted end line mean and SD result of reviewed articles which assessed the effect of motivational interviewing on Hgb. A1C and depressive symptoms among T2DM patients**

| **Authors & year** | **Hgb. A1C (Hemoglobin A1C)** | | | | | | **Depression symptoms** | | | | | |
| --- | --- | --- | --- | --- | --- | --- | --- | --- | --- | --- | --- | --- |
|  | **Experimental Group** | | | **Control Group** | | | **Experimental Group** | | | **Control Group** | | |
|  | **Mean** | **SD** | **N** | Mean | **SD** | **N** | **Mean** | **SD** | **N** | **Mean** | **SD** | **N** |
| Calhoun et al.(2010) | 8.969 | 1.69 | 20 | 8.8 | 1.8 | 26 | 7.18 | 7.68 | 20 | 11.65 | 11.04 | 26 |
| Celano et al.(2019) | 8.48 | 1.54 | 10 | 8.87 | 1.77 | 12 | 2.6 | 1.8 | 10 | 3.1 | 1.4 | 12 |
| Garry Welch et al.(2010) | 9.47 | 1.35 | 57 | 9.58 | 1.29 | 58 | NA | NA | NA | NA | NA | NA |
| Huang CY et al.(2016) | 6.16 | 0.92 | 31 | 7.49 | 1.82 | 30 | 13.99 | 3.68 | 31 | 22.42 | 3.56 | 30 |
| R.A. GABBAY et al.(2013) | 7.8 | 1.7 | 188 | 8.0 | 1.8 | 233 | 10 | 11 | 188 | 14 | 14 | 233 |
| S.M. Chen et al.(2012) | 8.16 | 1.73 | 104 | 8.48 | 1.78 | 110 | 6.57 | NA | 57 | 6.82 | NA | 58 |
| Stuckey et al. (2009) | 8.29 | 2.11 | 276 | 8.50 | 2.16 | 273 | 21.4 | 9.4 | 276 | 21.9 | 8.97 | 273 |

*SD: standard deviation, N: Total sample size*
