# Supplementary material for: Effect of motivational interviewing intervention on HgbA1C and depression in people with type 2 diabetes mellitus (systematic review and meta-analysis)
Source: PLoS One. 2020 Oct 23;15(10):e0240839. doi: 10.1371/journal.pone.0240839 (PMC7584232; doi:10.1371/journal.pone.0240839)
Supplement: Attachment — Submitted filename: RESPONSE TO CHIEF EDITOR.docx [file pone.0240839.s003.docx]

**RESPONSE TO CHIEF EDITOR**

_________________________________________________________________________

**In the reviewed article originally stated as:**

Aside from the staggering economic and medical consequences of diabetes, the psychological and quality-of-life costs of diabetes are also substantial **(Li, Ford, Strine, & Mokdad, 2008) (page number: 578).**

**Citation: *Pearce, Michelle J., Katherine Pereira, and Ellen Davis. "The psychological impact of diabetes: a practical guide for the nurse practitioner." Journal of the American Association of Nurse Practitioners 25.11 (2013): 578-583)***

**In the submitted manuscript stated as**

In addition to physical, economic and medical consequences of diabetes, the psychological and quality-of-life costs of diabetes are also substantial **(page number: “3”), ref. No: 8**

**Based on editors comment rephrased as:**

Diabetes has an effect on mental health and health related quality of life in addition to impact to body, finance and health care system **(page number: “3”) , ref. No: 8**

____________________________________________________________________________________

**In the reviewed article originally stated as:**

Motivational interviewing (MI), a patient-centered behavior change strategy, has proven valuable in the treatment of addictions and other chronic medical conditions. MI aims to identify and reduce patient ambivalence regarding health behavior change and to improve patient perceptions of the

importance of behavior change and confidence (self-efficacy) ***( Miller WR, Rollnick S. , 2002 and CDC, 2001)*** **( page number: 5). Ref. No: 8,9**

**Citation: Welch, Garry, et al. "Motivational interviewing delivered by diabetes educators: does it improve blood glucose control among poorly controlled type 2 diabetes patients?." Diabetes research and clinical practice 91.1 (2011): 54-60)**

**In the submitted manuscript stated as**

Motivational interviewing (MI) is a patient-centered behavior change strategy, has proven valuable in the treatment of addictions and other chronic medical conditions including diabetes. MI aims to find and reduce patient ambivalence about health behavior change and to improve patient perceptions of the importance of behavior change and confidence (self-efficacy) **(page number: “4”), ref. No: 17**

**Based on editors comment rephrased as:**

Motivational interviewing (MI) is client based way of life style modification which is supported by scientific evidences for management of substance abuse and other long-term illness like diabetes. Its goal is to identify and decrease patient inconsistency in health related activities and promote patients’ insight for advantage of healthy life style modification and self-reliance **(page number: “4”), ref. No: 17**

**In the reviewed article originally stated as:**

Effect of motivational interviewing on self-management in patients with type 2 diabetes mellitus: A meta-analysis. **(Page number: “291”),** this is title of the paper

**Citation: (Song D, Xu T-Z, Sun Q-H. Effect of motivational interviewing on self-management in patients with type 2 diabetes mellitus: a meta-analysis. International Journal of Nursing Sciences. 2014;1(3):291-7.)**

**In the submitted manuscript stated as**

Effect of MI on self-management in patients with none insulin dependent diabetes mellitus **(Page number: “4”), ref. 24**

**Based on editors comment rephrased as:**

Impact of motivational interviewing on self-care practices among clients with insulin dependent diabetes mellitus **(Page number: “4”), ref. 24**

**In the reviewed article originally stated as:**

Individual use of MI for different problems results in behavioral change and improvement of disease symptoms, which has been proven by several valid clinical trials **(Page number “3/6), ref. No** [36-38]

**Citation: Salimi, C., S. Momtazi, and S. Zenuzian. "A review on effectiveness of motivational interviewing in the management of diabetes mellitus." J Psychol Clin Psychiatry 5.4 (2016): 1-6.)**

**In the submitted manuscript stated as**

Several valid clinical trials proved that individual use of MI results in behavioral change and improvement of disease symptoms. **(Page number: “19”), ref. 46**

**Based on editors comment rephrased as:**

A number of qualified experimental studies evident that client who takes MI shows improvement on self-management as well as manifestations of illness. **(Page number: “19”), ref. 46**

**RESPONSE TO CHIEF EDITOR ON UPDATE OF ARTICLE SEARCH**

**Date 6/15/2020**

According to your previous email (on Mon, Jun 1, 10:16 AM) there was a request to **update the article search** for our review within range of **1/4/2019-12/6/2020**,

Therefore, we searched for the last two weeks to update our search using a title of effect of motivational interviewing intervention on Hgb. A1C value and depression in people with type 2 diabetes mellitus (T2DM)

The PICOS classifications used to guide our search were P: people with type 2 diabetes, I: MI interventions, C: usual care or a non-MI intervention, O: Hgb. A1C value and Depression and S: randomized controlled trials (RCTs) & pseudo RCT/ per-post intervention.

According to the above criteria we made a search on databases of Cochran Library, Google scholar, PubMed, MEDLINE Plus, Clinical Trials.gov.

Then **no article was found** in search of research articles and **no change was made on results of our reviewed article** (systematic review and meta-analysis).
