# Supplementary material for: Effect of motivational interviewing intervention on HgbA1C and depression in people with type 2 diabetes mellitus (systematic review and meta-analysis)
Source: PLoS One. 2020 Oct 23;15(10):e0240839. doi: 10.1371/journal.pone.0240839 (PMC7584232; doi:10.1371/journal.pone.0240839)
Supplement: Attachment — Submitted filename: Response to reviewers.docx [file pone.0240839.s004.docx]

A. **RESPONSE TO ACADEMIC EDITOR**
1. **Uploaded document documents::** As per your request documents such as response to reviewers (academic editor & academic reviewer), Revised Manuscript with & without track changes or highlights, supporting information (language edit) 1, “new manuscript” (language edited), data set as supporting information2 and updated cover letter were uploaded.
2. **Changes in financial disclosure:** Did not make any change in the financial disclosure because **“authors received no specific funding for this work.”**
3. **Laboratory protocols:** It is not applicable for this study because it is **a systematic review and meta-analysis** but up on your requested we can submit the **systematic review protocol/ proposal** which is developed according to Cochrane Handbook for Systematic Reviews of Interventions.
4. **Manuscript meets PLOS ONE's style requirements: w**e ensured that manuscript including table & figure were revised to meet PLOS ONE’s style requirement as per the suggested PLOS ONE style templates
5. **Language editing:** Our Manuscript was copyedit for language usage, spelling, and grammar by Dr. Mary Morgan from Columbia university, USA (email: [mbm4@cumc.columbia.edu](mailto:mbm4@cumc.columbia.edu)) and Dr. Carmen (email: [chenyta08@yahoo.com](mailto:chenyta08@yahoo.com)) from Mekelle university, Ethiopia. Uploaded as **Supporting Information file 1**
6. **Data availability:** Study’s data set (*extracted from reviewed studies on effect of MI intervention on HgbA1C and depressive symptoms among T2DM patients)* was uploaded as was Data set was uploaded as **Supporting Information files (using Table, excel, Stata & revman5) in the form of zipped file**
7. **Please upload a copy of Figure 7, to which you refer in your text on page 27 (18?):** It was an error in writing the No consecutively then corrected as per your comment ( Fig 7)... **page 22**
8. **Competing interest disclosure: The authors have declared that no competing interests exist for financial, personal**

**B. RESPONSE TO REVIEWERS**

1. **The aim of study is to reveal the effect of motivational interviewing (MI) intervention on depressive symptoms for patients with diabetes. However, author’s assessment for them is unclear (miss match between result and conclusion).**

- The finding of four studies in systematic review and pooled result of meta-analysis (fixed effect model analysis) identified that there was statistically significant improvement of depressive symptoms
- But the pooled result of meta-analysis in fixed effect model analysis had heterogeneity then further **subgroup analysis using random effect model was required** then **to get the true effect subgroup analysis according to MI session time and follow-up period was done.**
- There for pooled result of subgroup analysis showed that MI intervention is not effective for depressive symptoms ***(means do not have significant difference in the reduction of depressive symptoms among intervention and control group)***
- Then to avoid confusion results of systematic review and pooled result in fixed effect model analysis for depressive symptoms were removed**.**
- ***The pooled results of subgroup analysis rephrased in line to conclusion and in such away readers could understand as per your comment.***
- **Then Rephrased as “** Effect of MI intervention on depressive symptoms was identified through subgroup analysis according to intervention session time (30 or 60-80 minutes) and Follow-up period (3 or 24 months) then result showed that there was no significant difference in the reduction of depressive symptoms between the intervention and control groups. The output results were (WMD, -1.58; 95% CI, -5.05 to -0.188; p = 0.37; I2=48%), (WMD, -4.30; 95% CI, -9.32 to -0.73; p = 0.09; I2=95%) , (WMD, -4.45; 95% CI, -10.58 to 1.69; p= 0.16; I2=96%) and (WMD, -2.12; 95% CI, -5.54 to 1.30; p = 0.22; I2=83%) respectively”. **page 2 and 3 (Abstract ) and page 25**

2. **HgbA1C is unusual abbreviation. Motivation interviewing should be expressed as full spelling.**

- HgbA1C is written in usual way or expanded form as glycosylated hemoglobin A1C (HgbA1C) throughout the manuscript as per your comment and MI is also written in the expanded form as Motivation interviewing (MI) throughout the manuscript as per your comment

3. **What is the difference for NIDD and NIDDM ( Search strategy section: on page 7 and 8 )**

- Both are the same used to describe type 2 diabetes mellitus as none insulin dependent diabetes or diabetes mellitus and there is no difference. Those are key words or mesh terms we used to search articles for review because authors may use either of them interchangeably in their article as key word.

4. **I feel difficulty to see Table 1. Please reconsider to the structure of table1.**

- Table 3 **(page 15& 16)** font size increased or corrected to make it visible and Table 1 **(Page 9)** “Evaluation of the methodological quality of the reviewed studies (RCT) (6 in number) based on JBI appraisal checklist**.** Its column is reviewed articles **name of first author *(with reference number: 39,16,32,33, 34 and 43)*** and its raw is evaluation criteria (yes/No). Font size increased to make it visible

5. **Fig2, 3; Font type should be changed as the same as the others.**

- Because of the figures are analyses outputs of the software in the form of picture then copied and directly pest directly because of this **could not change or modify the font style** except increase its size. Therefore as the size of the picture or figure increase then font size also increased so that legible to the reader. Additionally the main important **pooled result were written properly as *(WMD, -0.27; 95% CI, -0.46 to -0.09; p= 0.004)* and *(WMD, -2.63; 95% CI, -3.45 to -1.80; p< 0.00001)*** respectively **(Page 19 & 20).** Moreover, as requirement of the journal figures are uploaded separately & individually in the form of TIFF

6. **Table 6; the font type for “Huang” is different for the others.**

- The font type for “Huang” which is differ for the others or made bold then corrected or made similar font type like others or avoid from making bold the word Huang **(page 20 & 22).**

7. **Ref 34 seems to include Chinese characteristics:** The Chinese character is removed **(Reference section)**
